# An Intracellular Ammonium Transporter Is Necessary for Replication, Differentiation, and Resistance to Starvation and Osmotic Stress in *Trypanosoma cruzi*

**DOI:** 10.1128/mSphere.00377-17

**Published:** 2018-01-17

**Authors:** Teresa Cruz-Bustos, Evgeniy Potapenko, Melissa Storey, Roberto Docampo

**Affiliations:** a Center for Tropical and Emerging Global Diseases, University of Georgia, Athens, Georgia, USA; b Department of Cellular Biology, University of Georgia, Athens, Georgia, USA; Indiana University School of Medicine

**Keywords:** *Trypanosoma cruzi*, amino acids, ammonia, ammonium, autophagy, lysosomes, reservosomes

## Abstract

*Trypanosoma cruzi*, the etiologic agent of Chagas disease, undergoes drastic metabolic changes when it transits between a vector and mammalian hosts. Amino acid catabolism leads to the production of ammonium (NH_4_^+^), which needs to be detoxified. However, *T. cruzi* does not possess a urea cycle, and it is unknown how intracellular levels of ammonium are controlled. In this work, we identified an intracellular ammonium transporter of *T. cruzi* (TcAMT) that localizes to acidic compartments (reservosomes, lysosomes). TcAMT has 11 transmembrane domains and possesses all conserved and functionally important amino acid residues that form the pore in other ammonium transporters. Functional expression in *Xenopus* oocytes followed by a two-electrode voltage clamp showed an inward current that is NH_4_^+^ dependent at a resting membrane potential (*V*_*h*_) lower than −120 mV and is not pH dependent, suggesting that TcAMT is not an NH_4_^+^/H^+^ cotransporter but an NH_4_^+^ or NH_3_/H^+^ transporter. Ablation of *TcAMT* by clustered regularly interspaced short palindromic repeat analysis with Cas9 (CRISPR-Cas9) resulted in significant defects in epimastigote and amastigote replication, differentiation, and resistance to starvation and osmotic stress.

**IMPORTANCE** *Trypanosoma cruzi* is an important human and animal pathogen and the etiologic agent of Chagas disease. The parasite undergoes drastic changes in its metabolism during its life cycle. Amino acid consumption becomes important in the infective stages and leads to the production of ammonia (NH_3_), which needs to be detoxified. We report here the identification of an ammonium (NH_4_^+^) transporter that localizes to acidic compartments and is important for replication, differentiation, and resistance to starvation and osmotic stress.

## Abstract (precis)

*Trypanosoma cruzi* is an important human and animal pathogen and the etiologic agent of Chagas disease. The parasite undergoes drastic changes in its metabolism during its life cycle. Amino acid consumption becomes important in the infective stages and leads to the production of ammonia (NH_3_), which needs to be detoxified. We report here the identification of an ammonium (NH_4_^+^) transporter that localizes to acidic compartments and is important for replication, differentiation, and resistance to starvation and osmotic stress.

## INTRODUCTION

*Trypanosoma cruzi* is the etiologic agent of American trypanosomiasis (Chagas disease), a disease causing considerable morbidity and mortality in Latin America. *T. cruzi* has a complex life cycle involving two forms present in its insect vector, the replicative epimastigote that inhabits the intestine and the nonreplicative and infective metacyclic trypomastigote present in feces and urine, and two forms present in its mammalian host, the replicative intracellular amastigote and the nonreplicative bloodstream trypomastigote.

Although most metabolic studies have been done with the epimastigote stage (1), which can easily be grown in axenic culture, genomic data (2) and proteomic information of *T. cruzi*’s different stages (3) have facilitated studies of the enzymatic pathways present in this parasite. Recent metabolomics studies (4) have revealed that epimastigotes transitioning from the exponential to the stationary phase switch from glucose to amino acid consumption as a source of energy. Amino acid catabolism, together with fatty acid oxidation (3), can also be important in the intracellular stages. Amino acid catabolism leads to the production of energy as well as the nitrogenous waste product ammonia (NH_3_), which arises from the amine groups and needs to be excreted to avoid toxicity. Ammonia is in a pH-dependent equilibrium with ammonium (NH_4_^+^) and has a pK_a_ of 9.25, most of which (>98%) is in the charged form at physiological pH (5). Ammonium increases when epimastigotes are submitted to hyposmotic stress as a result of increased amino acid catabolism and becomes sequestered in acidic organelles (6), while the reverse occurs when these cells are submitted to hyperosmotic stress (7). These changes are important for decreasing or increasing, respectively, the levels of amino acids that act as compatible osmolytes during osmotic stress and help in maintaining the cytosolic ionic strength despite changes in the water content of the cells (7).

In many eukaryotic cells, ammonia is detoxified through the Krebs-Henseleit, or urea, cycle. However, the urea cycle is absent in trypanosomatids (8). One way to control levels of ammonia in the cell is through incorporation into amino acids like glutamine or asparagine by glutamine (9) and asparagine (10) synthetases, respectively. Another way might be through its excretion or storage in intracellular compartments by ammonium transporters. In this work, we report the presence of a *T. cruzi* intracellular ammonium transporter (TcAMT) in acidic compartments, which we characterized by electrophysiology after its expression in *Xenopus laevis* oocytes. Ablation of *TcAMT* by clustered regularly interspaced short palindromic repeat analysis (CRISPR) with CRISPR-associated protein 9 (Cas9) led to significant defects in epimastigote growth, metacyclogenesis, amastigote replication, and resistance to starvation and osmotic stress.

## RESULTS

### *TcAMT* sequence analysis.

The *T. cruzi* genome contains a single gene, *TcAMT*, that belongs to the ammonium transporter/methylammonium permease/rhesus protein (Amt/Mep/Rh) family of ammonium transporters. No orthologs are present in the genomes of *Trypanosoma brucei* or *Leishmania* spp. The highest BLASTp matches were identified as ammonium transporters from *Archaeoglobus fulgidus*, *Escherichia coli*, and *Candida albicans* (35 to 39% identities). Hydropathy analysis revealed a profile very similar to those of other ammonium transporters with 11 transmembrane domains. The protein sequence shares other conserved characteristics of ammonium transporters. It consists of 514 amino acids, has an apparent molecular mass of 55.5 kDa, has a pI of 6.72, and possesses all 14 conserved and functionally important amino acid residues that form the pore of ammonium transporters (11) (see Fig. S1 in the supplemental material [red]).

10.1128/mSphere.00377-17.1 FIG S1  Alignment of AMT amino acid sequences. The 14 residues reported to be of functional significance for conducting ammonium through the pore region are indicated in red. The 11 hydrophobic regions are indicated in blue. The following sequences with the indicated accession numbers were obtained from the GenBank database: *Geosiphon pyriformis* (GpAMT1; JX535577); *Escherichia coli* (EcAMT; WP_001477975.1); *Candida albicans* (CaAMT; EEQ45414.1); *Trypanosoma cruzi* (TcAMT; XP_811961); *Archaeoglobus fulgidus* (WP_010878477.1). Residues labeled with asterisks are identical. Download FIG S1, TIF file, 2.3 MB. Copyright © 2018 Cruz-Bustos et al. 2018 Cruz-Bustos et al. This content is distributed under the terms of the Creative Commons Attribution 4.0 International license.

### *TcAMT*** overexpression.**

**FIG 1:**
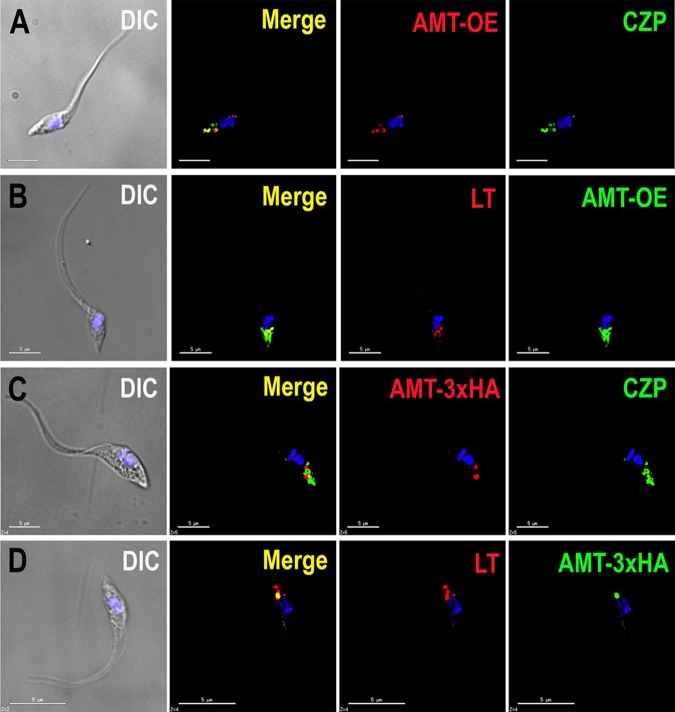
Localization of TcAMT in epimastigotes. (A) Overexpressed TcAMT (AMT-OE) detected with anti-HA antibodies (red) colocalizes with anticruzipain antibodies (green). (B) Overexpressed TcAMT (green) colocalizes with LysoTracker (LT; red). (C) Endogenously tagged TcAMT (AMT-3×HA; red) does not colocalize with anticruzipain (green). (D) Endogenously tagged TcAMT (green) colocalizes with LysoTracker (red). DIC, differential interference contrast microscopy; CZP, cruzipain. Yellow indicates merged results. DAPI staining appears in blue. (A to D) Bars = 5 µm.

We generated a cell line overexpressing *TcAMT* (*TcAMT*-OE cells) that was cloned in the pTREX-n vector, as described in Materials and Methods. In epimastigotes, overexpressed TcAMT colocalized with antibodies against cruzipain, which is a protease localized to reservosomes (12), and TcAMT was found in additional vacuoles that were not labeled with antibodies to cruzipain (Fig. 1A). However, there was a perfect colocalization of *TcAMT*-OE with LysoTracker Red DND-99, suggesting that all *TcAMT*-OE-labeled compartments are acidic (Fig. 1B).

### TcAMT** C-terminal endogenous tagging.**

We also generated a cell line with endogenously tagged TcAMT using the CRISPR-Cas9 technique that we recently described (13). Interestingly, TcAMT endogenously tagged with three hemagglutinin tags (TcAMT-3×HA) did not colocalize with cruzipain but labeled other vacuoles (Fig. 1C). However, TcAMT3×HA colocalized with LysoTracker Red DND-99 (Fig. 1D), a dye used to label acidic compartments.

### TcAMT localization in infective stages.

**FIG 2:**
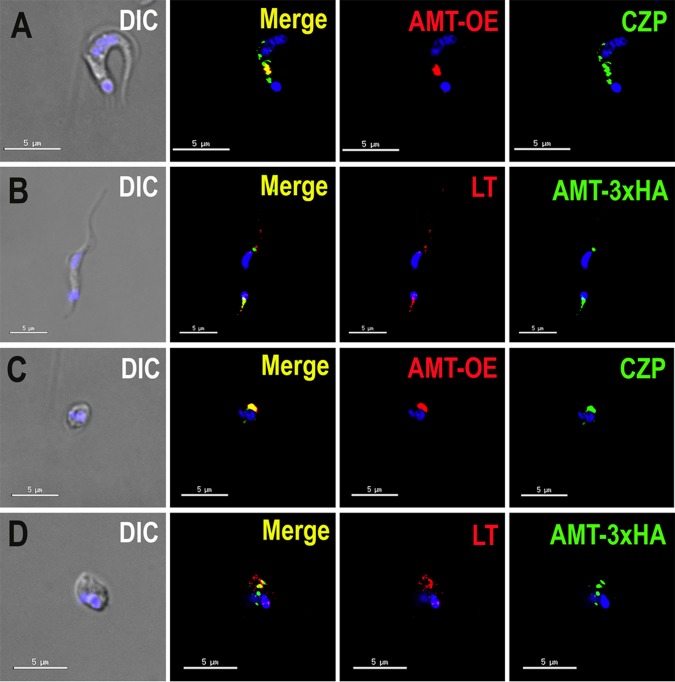
Localization of TcAMT in infective stages. (A) *TcAMT*-OE detected with anti-HA antibodies (red) partially colocalizes with anticruzipain antibodies (green) in trypomastigotes. (B) Endogenously tagged TcAMT (AMT-3×HA; green) colocalizes with LysoTracker (LT; red) in trypomastigotes. (C) *TcAMT*-OE (red) colocalizes with anticruzipain (green) in amastigotes. (D) Endogenously tagged TcAMT (green) colocalizes with LysoTracker (red) in amastigotes. DIC, differential interference contrast microscopy. DAPI staining appears in blue. (A to D) Bars = 5 µm.

To investigate the localization of TcAMT in the infective stages, we induced the differentiation of *TcAMT*-OE and TcAMT-3×HA epimastigotes into infective metacyclic trypomastigotes (metacyclogenesis) by incubating them in triatome artificial urine (TAU) medium as described under Materials and Methods and then infected Vero cells. After infection, we collected trypomastigotes and amastigotes and performed immunofluorescence analyses. Figure 2A and C show that overexpressed TcAMT colocalized with cruzipain, although cruzipain labeled additional structures in trypomastigotes (Fig. 2A). There was also colocalization of TcAMT-3×HA with LysoTracker Red DND-99 (Fig. 2B and D).

### Protein expression after hyposmotic stress.

**FIG 3:**
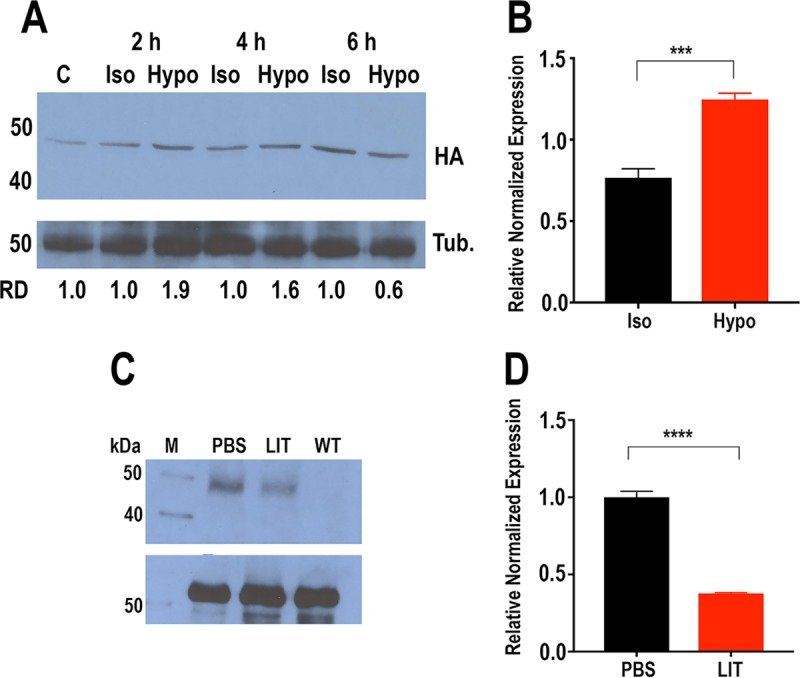
Protein and mRNA expression after hyposmotic stress. (A) Western blot analysis of *TcAMT*-OE epimastigotes maintained under isosmotic (Iso) or hyposmotic (Hypo) conditions for up to 6 h using antibodies against HA. Reaction mixtures with antitubulin antibodies (Tub.) were used as a loading control (lower panel). Molecular weight markers are shown on the left. Relative densities (RD) compared to those of the corresponding isosmotic control are shown below the blot. Lane C is time 0. (B) qRT-PCR of epimastigotes maintained under isosmotic or hyposmotic conditions for 30 min. (C) Western blot analysis of *TcAMT*-OE epimastigotes maintained in PBS or LIT medium for 20 h using antibodies against HA. Antitubulin antibodies were used as a loading control (lower panel). Molecular mass markers (lane M) are shown on the left. WT, wild type. (D) qRT-PCR of epimastigotes maintained in PBS or LIT medium for 20 h. Values are means ± standard deviations (SD) (*n* = 3). ***, *P* < 0.001; ****, *P* < 0.0001 by two-way analysis of variance (ANOVA).

Western blot analysis of *TcAMT*-OE epimastigotes using antibodies against its HA tag detected a band of the expected size (Fig. 3A). In agreement with the reported ammonium increase after hyposmotic stress (6), *TcAMT*-OE protein expression increased after epimastigotes were subjected to hyposmotic stress, with higher expression at 2 and 4 h (Fig. 3A). This was paralleled by an increase in mRNA expression, as detected by quantitative reverse transcription-PCR (qRT-PCR) (Fig. 3B). TcAMT overexpression also increased after epimastigotes were starved in phosphate-buffered saline (PBS) for 20 h compared to levels in cells maintained in liver infusion tryptose (LIT) medium (Fig. 3C), and these changes were also detected at the mRNA level (Fig. 3D).

### Functional expression of *TcAMT* in *Xenopus laevis* oocytes and basic membrane transport characteristics.

**FIG 4:**
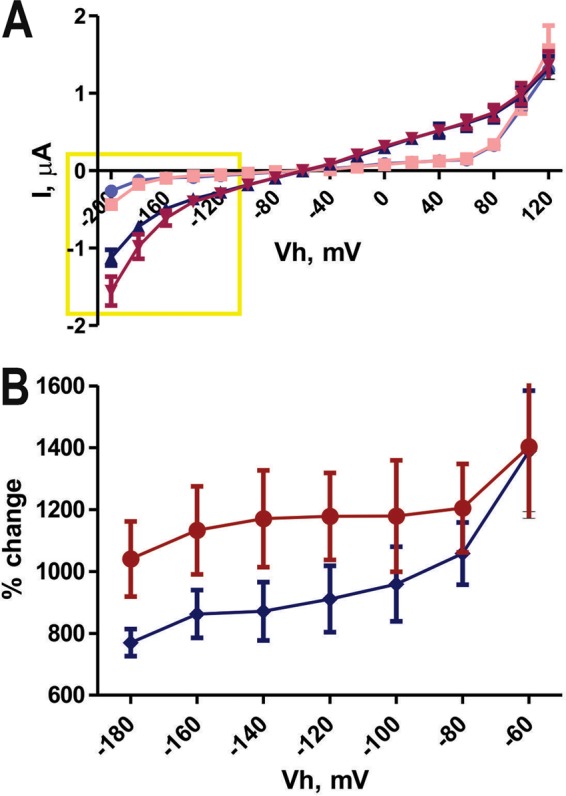
Ammonium-induced currents in *TcAMT*-expressing oocytes. (A) Average step protocol data recorded in oocytes injected with *TcAMT* cRNA (red and pink) and DEPC (blue and light blue) in the presence of 10 mM NH_4_^+^ (red and blue) and in regular ND96 solution (pink and light blue) (I, intensity); (B) same data (most hyperpolarized part of curve) normalized as a percentage of the current amplitude increase in the presence of ammonium in control oocytes (blue) and those expressing *TcAMT* (red).

Healthy *Xenopus laevis* oocytes with resting membrane potentials (*V*_*h*_s) lower than −30 mV and leak currents of less than 50 nA were used. We first analyzed the voltage dependency of the transporter by registering the currents generated in control oocytes injected with diethylpyrocarbonate (DEPC) and those expressing *TcAMT* in response to a voltage step change in *V*_*h*_ from −180 to +120 mV under control conditions and in the presence of ammonium. The current amplitude was measured at the stable steady-state part of the traces. Ten millimolar NH_4_^+^ induces independent elevation of both inward and outward currents in control and *TcAMT*-expressing oocytes, indicating the presence of endogenous currents stimulated by ammonium. Quantification of averaged currents in both control and *TcAMT*-expressing oocytes (Fig. 4A) in regular ND96 solution and in the presence of 10 mM NH_4_^+^ showed a significant increase in the steady-state current in *TcAMT*-expressing oocytes compared with that in controls (Fig. 4B; data were normalized to those in Fig. 4A) at a *V*_*h*_ more negative than −120 mV (up to −180 mV).

**FIG 5:**
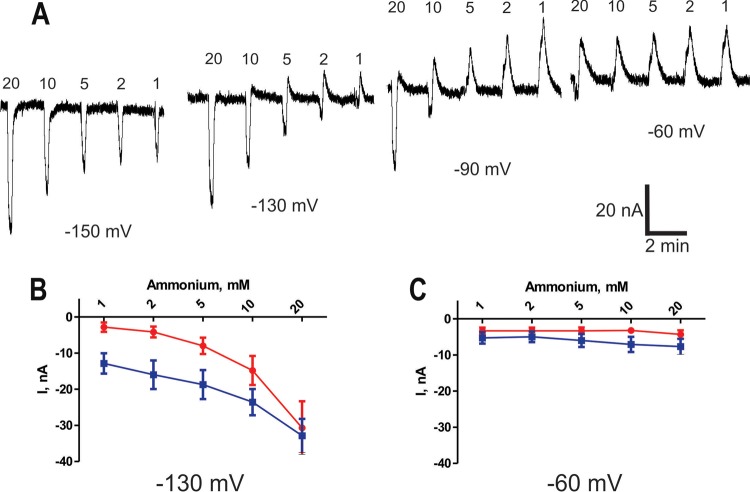
Voltage and ammonium concentration dependence of TcAMT. (A) Inward and outward current transients induced by brief (10-s) application of NH_4_^+^ at different concentrations (1, 2, 5, 10, and 20 mM) and *V*_*h*_s (−60, −90, −130, and −150 mV); (B, C) averaged amplitudes of transients induced by application of NH_4_^+^ in different concentrations recorded at *V*_*h*_s equal to −130 mV (B) and −60 mV (C).

Figure 5A shows the currents generated at various holding potentials (*V*_h_ = −150 to −60 mV) by addition of different millimolar concentrations of NH_4_^+^ to oocytes expressing *TcAMT*. The inward current amplitude at a holding potential of −150 mV increased in an NH_4_^+^ concentration-dependent manner from 1 to 20 mM. The results indicate transport of a positive charge into the cell. Current changes were significantly different from those observed in control oocytes (Fig. 5B), probably related to endogenous transporting mechanisms. At holding potentials between −130 and −60 mV, the currents changed to outward currents without a significant NH_4_^+^ dependence (Fig. 5A and C).

**FIG 6:**
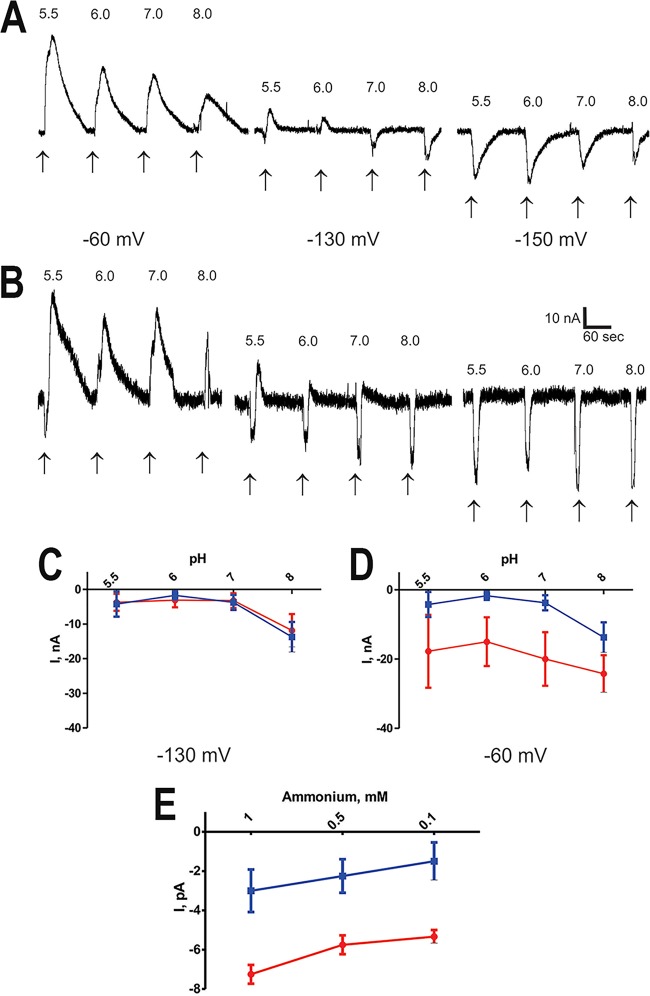
pH dependence of TcAMT. (A, B) Inward and outward current transients induced by brief (10-s) application of 10 mM NH_4_^+^ at different pHs (5.5, 6.0, 7.0, and 8.0) and *V*_*h*_s (−60, −130, and −150 mV) in DEPC (A)- and *TcAMT* cRNA (B)-injected oocytes; (C) averaged amplitudes of transients induced by application of NH_4_ at different pHs recorded at *V*_*h*_s equal to −130 mV and −60 mV; (D) dose dependence of currents recorded at a *V*_*h*_ of −120 mV in the presence of low concentrations of ammonium (1, 0.5, and 0.1 mM) in DEPC (blue)- and *TcAMT* cRNA (red)-injected oocytes.

Figure 6A to D show that the NH_4_^+^-dependent inward current (−130 to −150 mV) showed no dependence on the pH of the extracellular medium from pH 5.5 to 8, while the NH_4_^+^-insensitive outward current (−60 mV) showed a pH dependence when the extracellular pH was changed to acidic values. Figure 6E shows that inward currents at a holding potential of −120 mV were also dose dependent when concentrations of ammonium of 1 mM or lower were used.

Taken together, the results indicate the detection of two distinctive overlapping transient currents at different *V*_*h*_s: outward (dominant at −60 and −90 mV) and inward (dominant at less than −130 mV). The outward current is voltage dependent, gradually increasing when *V*_*h*_ is more positive, is pH dependent, and is insensitive to NH_4_^+^ and may be due to activation of endogenous Na^+^/H^+^ of K^+^/H^+^ exchangers. The inward current is voltage dependent, increasing when *V*_*h*_ is more negative, is NH_4_^+^ dependent at *V*_*h*_s lower than −120 mV, and is not pH dependent, suggesting that TcAMT is not an NH_4_^+^/H^+^ cotransporter but an NH_4_^+^ transporter.

### Ablation of *TcAMT* in epimastigotes.

**FIG 7:**
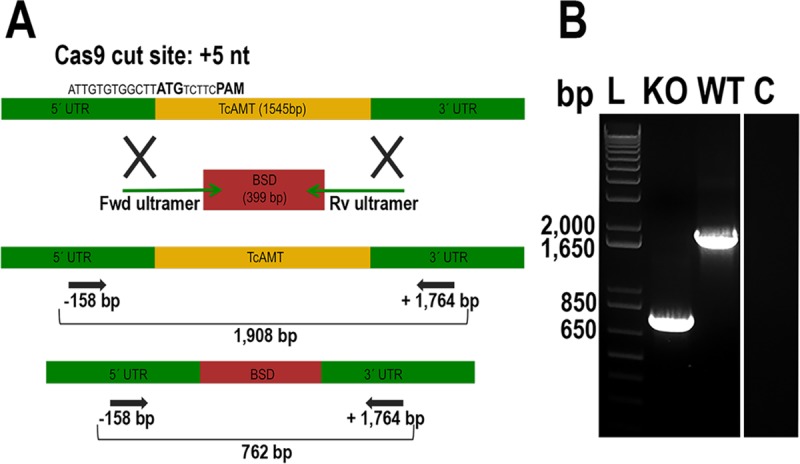
*TcAMT* knockout cells. (A) Schematic representation of the strategy used to generate a *TcAMT*-KO mutant by CRISPR-Cas9-induced homologous recombination. A double-stranded genomic DNA (gDNA) break was produced by Cas9 at nucleotide (nt) 5 of the *TcAMT* ORF. DNA was repaired with a blasticidin S-deaminase (BSD) cassette containing ~500-bp homologous regions from the *TcAMT* locus. Arrows show primers that were used to verify gene deletion by PCR. 5′ and 3′ UTR, 5′ and 3′ untranslated regions, respectively; Fwd, forward; Rv, reverse; PAM, photospacer adjacent motif. (B) PCR analysis using gDNA isolated from wild-type (WT) and *TcAMT*-KO (KO) cell lines. The intact locus generates a PCR product of 1,908 bp, while the replaced locus generates a fragment of 762 bp. Lane C, PCR negative control; lane L, molecular ladder. The control was from the same gel and is separated because duplicate samples of the* TcAMT*-KO cell lines that were in the original gel were deleted.

We used the CRISPR-Cas9 system with a DNA donor cassette for DNA repair to knock out *TcAMT* (Fig. 7A) in epimastigotes. Five weeks of selection with G418 (to select for the p-TREX vector with the single-guide RNA [sgRNA] and Cas9) and blasticidin (to select for the DNA donor) resulted in resistant populations with *TcAMT* deleted. As shown in Fig. 7B, *TcAMT* was ablated and replaced by the blasticidin resistance gene in *TcAMT*-knocked-out (*TcAMT*-KO) cells.

### Phenotypic changes in *TcAMT*-KO cells.

**FIG 8:**
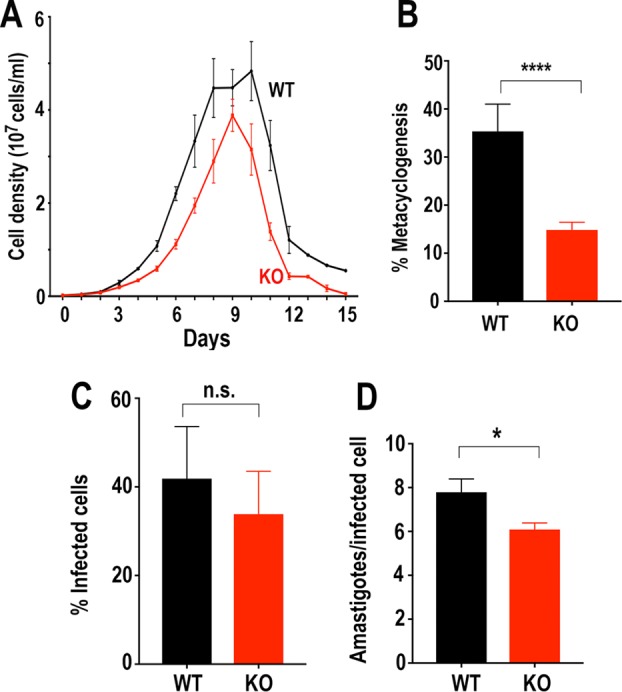
Phenotypic changes in *TcAMT*-KO mutant epimastigotes. (A) Growth of wild-type (WT) and *TcAMT*-KO epimastigotes in LIT medium. Values are means ± SD (*n* = 3), and differences are significant from 3 to 15 days. ***, *P* < 0.001 by Student’s paired *t* test. (B) Percentages of metacyclic trypomastigotes in epimastigote cultures after incubation in TAU 3AAG medium. (C) Effect of *TcAMT* knockout on trypomastigote infection of Vero cells. There were no significant differences in percentages of infected Vero cells. (D) Effect of *TcAMT* knockout on amastigote replication after 48 h. Differences were significant. (B to D) Values are means ± SD (*n* = 3, 5, and 4, respectively). n.s., no significant difference. ****, *P* < 0.0001; *, *P* < 0.05 by one-way and two-way ANOVA with multiple comparisons.

Growth of epimastigotes in LIT medium was significantly reduced in *TcAMT*-KO cells (Fig. 8A). *TcAMT-*KO epimastigotes were able to differentiate into metacyclic trypomastigotes at a lower proportion than that of wild-type cells (Fig. 8B). The *TcAMT*-KO metacyclic trypomastigotes were able to infect host cells. After several cycles of infection to obtain enough culture-derived trypomastigotes, we performed *in vitro* infection assays to find the percentage of infected cells after incubation with *TcAMT*-KO trypomastigotes. Figure 8C shows that there was no significant difference between infections with wild-type and *TcAMT-*KO trypomastigotes. However, Fig. 8D shows that replication of amastigotes was significantly affected by *TcAMT* knockout, demonstrating that *TcAMT* is essential for normal intracellular replication.

**FIG 9:**
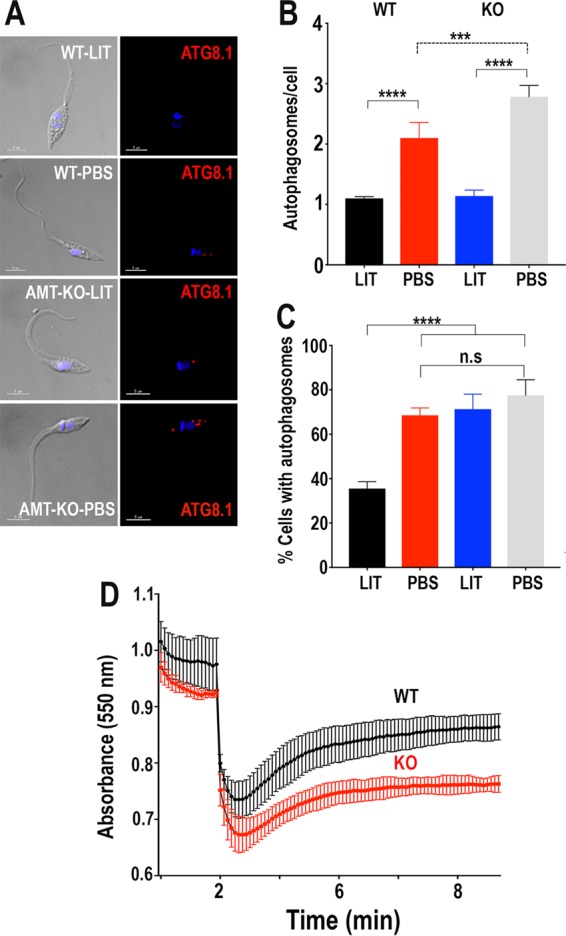
Autophagy in TcAMP-KO mutant epimastigotes. (A) Representative fluorescence microscopy images of wild-type (WT) and *TcAMT*-KO (AMT-KO) epimastigotes labeled with anti-TcATG8.1 antibody after incubation in LIT medium or PBS for 20 h. DAPI staining and DIC images are also shown. Bars = 5 µm. (B) Numbers of autophagosomes per cell under the different conditions shown in panel A. (C) Percentages of cells with autophagosomes under the conditions shown in panel A. (D) Regulatory volume decreases in epimastigotes. Cells suspended in isosmotic buffer were diluted with water to a final osmolarity of 150 mosM after 2 min, and relative changes in cell volume were monitored by determining absorbance at 550 nm as described in Materials and Methods. All experiments were done with the same amount of cells. Values are means ± SD from three independent experiments and are expressed as arbitrary absorbance units. Values are means ± SD (*n* = 3). ***, *P* < 0.001; ****, *P* < 0.0001 by one-way and two-way ANOVA with multiple comparisons.

We also evaluated starvation-induced autophagy in these cells by incubating them in PBS or LIT medium for 20 h. Immunofluorescence microscopy was done using antibodies against the autophagy marker Atg8.1, which is the ortholog of LC3-II in mammalian cells (14). *TcAMT*-KO cells had significantly increased numbers of autophagosomes per cell under starvation conditions (PBS incubation) (Fig. 9A and B). *TcAMT*-KO cells also had higher percentages of cells with autophagosomes when they were incubated in LIT medium, but the percentages of cells with autophagosomes were comparable to those for wild-type cells under starvation conditions (PBS incubation) (Fig. 9C). Finally, as NH_4_^+^ was found to have a role in osmoregulation, we investigated whether *TcAMT-*KO cells were deficient in their regulatory volume decrease after hyposmotic stress. Figure 9D shows that when mutant cells were subjected to a 50% reduction in osmolarity (from 300 to 150 mosM), swelling was more pronounced and recovery was slower than in control cells.

## DISCUSSION

We report here that *TcAMT* encodes an NH_4_^+^ transporter that localizes to acidic compartments of *T. cruzi*. The transporter is important for *in vitro* epimastigote growth, metacyclogenesis, amastigote replication, and resistance to starvation and osmotic stress.

With the exception of *Dictyostelium discoideum* (15), the fungus *Geosiphon pyriformis* (16), and plants (11), which have intracellular NH_4_^+^ transporters, most organisms have plasma membrane-associated NH_4_^+^ transporters. *D. discoideum* possesses three NH_4_^+^ transporters (AmtA, AmtB, and AmtC), two of which localize to the membranes of contractile vacuoles (AmtB) and to the membranes of endolysosomes and phagosomes (AmtA, AmtB), while the NH_4_^+^ transporters of *G. pyriformis* and plants are in storage vacuoles. All these intracellular compartments are acidic.

*T. cruzi* is one of the few trypanosomatids whose genome sequence is known that possesses an NH_4_^+^ transporter. No orthologs are found in *T. brucei* or *Leishmania* spp. Interestingly, when overexpressed, TcAMT colocalizes with cruzipain in epimastigotes, a marker of reservosomes, a prelysosomal compartment previously characterized in epimastigotes (12, 17). However, endogenously tagged TcAMT localizes to acid compartments labeled with LysoTracker Red but not with cruzipain. It is possible that this localization in epimastigotes corresponds to the lysosomes and that when overexpressed, the transporter is accumulated in reservosomes. In this regard, we have reported before (13) that overexpression of proteins might affect their localization. Both *TcAMT*-OE and endogenously tagged TcAMT (TcAMT-3×HA) partially colocalize with cruzipain and LysoTracker Red in amastigotes and trypomastigotes.

When we expressed *TcAMT* in *Xenopus* oocytes, we detected two distinctive overlapping transient currents at different *V*_*h*_s: outward (dominant at − 60 and −90 mV) and inward (dominant at less than −130 mV). The inward current is NH_4_^+^ dependent at a *V*_*h*_ lower than −120 mV and is not pH dependent, suggesting that TcAMT is not an NH_4_^+^/H^+^ cotransporter but an NH_4_^+^ (or NH_3_/H^+^) transporter. The lack of pH dependence is in agreement with previous reports on members of the AMT/Mep/Rh family of transporters (18–20).

The phenotypic changes that occur in *TcAMT*-KO mutants are in agreement with a role for this transporter in survival under starvation and osmotic stress. When epimastigotes are submitted to hyposmotic stress, ammonium generation increases as a result of increased amino acid catabolism and acidic organelles are alkalinized, suggesting its transport to these vacuoles (6). In contrast, inhibition of amino acid catabolism when these cells are submitted to hyperosmotic stress leads to a decrease in ammonium levels (7). These changes result in a decrease or increase, respectively, in the levels of amino acids that act as compatible osmolytes during osmotic stress and help in maintaining the cytosolic ionic strength despite changes in the water content of the cells (7). On the other hand, an increase in protein consumption during starvation leads to increased production of ammonium and enhanced autophagy.

In summary, TcAMT is an intracellular NH_4_^+^ transporter involved in detoxification of this product of amino acid metabolism through its storage in acidic compartments. Its ablation results in significant alterations in replication and in the ability of the cells to differentiate and respond to starvation and osmotic stress.

## MATERIALS AND METHODS

### Chemicals and reagents.

Rabbit polyclonal antibody against cruzipain was a gift from Vanina Alvarez (Universidad Nacional de San Martin, Argentina), and the pMOTag4H vector was a gift from Thomas Seebeck (University of Bern, Bern, Switzerland). The Q5 hot-start high-fidelity DNA polymerase, BenchMark protein ladder, and Alexa Fluor-conjugated secondary antibodies were purchased from Invitrogen. Rat HA antibody was from Roche. The primers were purchased from Integrated DNA Technologies, Inc. The Magic Mark XP Western protein standard, Bio-Rad.IScript cDNA synthesis kit, CFX96 Touch real-time PCR detection system, and iQ SYBR Green supermix were from Bio-Rad. TRI Reagent and all other reagents of analytical grade were from Sigma-Aldrich.

### Cell culture.

*T. cruzi* Y strain epimastigotes were cultured in liver infusion tryptose (LIT) medium containing 10% heat-inactivated fetal bovine serum at 28°C. The overexpressing cell line was maintained in medium containing 250 μg/ml G418. The endogenously tagged cell line was maintained in medium containing 250 μg/ml G418 and 200 μg/ml hygromycin, and the medium of the knockout cell line contained 250 μg/ml G418 and 10 μg/ml blasticidin. Metacyclogenesis was obtained as described previously (21). Tissue culture cell-derived trypomastigotes and amastigotes were obtained from Vero cells infected with metacyclic trypomastigotes of *TcAMT-*OE and wild-type parasites. Metacyclic trypomastigotes were obtained by incubating epimastigotes in triatomine artificial urine (TAU) medium as described previously (21). We determined the growth rate of epimastigotes by counting cells in a Neubauer chamber. *In vitro* infection assays were done exactly as described before (21).

### Generation of the *TcAMT-*OE cell line.

Epimastigotes were transfected with the pTREX-n vector (22, 23) containing the *TcAMT* gene (EuPath accession number TcCLB.508317.50) with a hemagglutinin (HA) tag for localization and gain-of-function analysis. The construct for *TcAMT* overexpression was made as follows. The full sequence of *TcAMT* was PCR amplified with primers 1 and 2 (see Table S1 in the supplemental material) and *T. cruzi* DNA as the template. The amplified fragment was digested with XbaI and HindIII restriction enzymes and cloned into a pTREX-n vector that includes an HA epitope tag coding sequence, allowing the detection of the overexpressed protein with anti-HA antibody. Correct insertion was determined by PCR and sequencing.

10.1128/mSphere.00377-17.2 TABLE S1  Primers used in this work. Download TABLE S1, DOCX file, 0.1 MB. Copyright © 2018 Cruz-Bustos et al. 2018 Cruz-Bustos et al. This content is distributed under the terms of the Creative Commons Attribution 4.0 International license.

### Endogenous C-terminal tagging of *TcAMT* by CRISPR-Cas9.

To obtain the C-terminal tagging of *TcAMT*, we used the Cas9/pTREX-n vector that we developed for *T. cruzi* (24) to clone a specific single-guide RNA (sgRNA) sequence targeting the 3′ end of the gene. We cotransfected the 3′-end-tagged sgRNA/Cas9/pTREX-n construct with the specific DNA donor cassette amplified from the pMOTag-4H vector to induce homology-directed repair and to insert a specific tag sequence (3×HA) at the 3′ end of the gene, as described previously using primers 3 to 7 (24).

### Generation of *TcAMT-*KO cells.

A chimera sgRNA sequence to target the *TcAMT* gene was PCR amplified from plasmid pUC_sgRNA, containing the sgRNA backbone sequence (82 bp). One specific protospacer was included in forward primer 8, which, together with reverse primer 9 (Table S1), was used for sgRNA amplification. These primers also contained a BamHI restriction site for cloning into Cas9/pTREX-n upstream of the HX1 transsplicing signal to generate the *TcAMT*-sgRNA/Cas9/pTREX-n construct. The DNA donor cassette designed to promote homologous directed repair and ablation of the target gene after the double-strand break induced by Cas9 was obtained using a recombinant PCR strategy using primers 10 and 11 (Table S1), as described previously (21, 24). Gene deletion was verified by PCR (primers 7 and 12).

### Cell transfection.

*T. cruzi* epimastigotes were grown to a density of 1 × 10^7^ to 2 × 10^7^ cells/ml, washed once with cold PBS, pH 7.4, and resuspended in ice-cold Cytomix (120 mM KCl, 0.15 mM CaCl_2_, 10 mM K_2_HPO_4_, 2 mM EDTA, 5 mM MgCl_2_, 25 mM HEPES, pH 7.6) containing 25 µg of each DNA construct in 4-mm electroporation cuvettes. Three pulses (1.5 kV, 50 µF) were delivered by a Gene Pulser Xcell (Bio-Rad), with allowance of at least 10 s for recovering on ice, and then the cuvettes were incubated at room temperature for 15 min. Transfectants were cultured in 5 ml LIT medium supplemented with 20% fetal bovine serum (FBS), and after 24 h, the appropriate antibiotic (250 µg/ml G418 or 200 µ/ml hygromycin) was added. Stable cell lines were established and maintained under drug selection with appropriate antibiotics.

### SDS-PAGE and Western blot analyses.

Western blot analyses were performed using standard procedures (13). Thirty micrograms of protein from each cell lysate was mixed with 6× Laemmli sample buffer and analyzed by SDS-PAGE. Electrophoresis was performed as described previously (13). Electrophoresed proteins were transferred to nitrocellulose membranes using a Bio-Rad Transblot apparatus for 1 h at 100 V under cold conditions. Following transfer, the membrane blots were blocked with 5% nonfat dry milk in PBS containing 0.1% (vol/vol) Tween 20 (PBS-T) at room temperature for 1 h. Blots were probed with anti-HA rat monoclonal antibody (1:1,000 dilution) and anti-α-tubulin mouse antibody (1:40,000) for 1 h. The membranes were washed three times with PBS-T, and Western blot images were processed and analyzed using the Odyssey infrared system software (LICOR Biosciences).

### Immunofluorescence analysis.

Epimastigotes in log phase, trypomastigotes, and amastigotes were washed once with PBS at room temperature and fixed with 4% paraformaldehyde in PBS for 30 min at room temperature. The cells were adhered to poly-l-lysine-coated coverslips and then permeabilized for 3 min with 0.3% Triton X-100. Permeabilized cells were blocked with PBS containing 3% bovine serum albumin (BSA), 1% fish gelatin, 50 mM NH_4_Cl, and 5% goat serum for 1 h at room temperature. Then, cells were incubated with the primary antibody (rat anti-HA tag, 1:100; rabbit anti-cruzipain, 1:750) diluted in PBS (pH 7.4) for 1 h at room temperature. The cells were washed three times with PBS (pH 7.4) and then incubated for 1 h at room temperature in the dark with Alexa Fluor 488-conjugated goat anti-rabbit or Alexa Fluor 546-conjugated goat anti-rat antibody (both at 1:1,000). Following incubation with the secondary antibody, the cells were washed five times in PBS and once in water and then mounted on slides. Epimastigotes were treated with Lysotracker (0.5 μM) for 30 min at room temperature and dark environment and fixed as previously described. DAPI (4′,6-diamidino-2-phenylindole; 5 μg/ml) was included in the Fluoromount-G mounting medium to stain DNA. Control experiments were performed as described above but in the absence of primary antibody. Differential interference contrast and fluorescence optical images were captured with a 100× objective (1.35-aperture) lens under nonsaturating conditions in an Olympus IX-71 inverted fluorescence microscope with a Photometrix CoolSnapHQ charge-coupled device camera driven by DeltaVision software (Applied Precision, Issaquah, WA); images were then deconvolved for 15 cycles using Sotwarx deconvolution software.

### qRT-PCR.

Total RNA was isolated from wild-type epimastigotes using the TRI Reagent by following the manufacturer’s instructions. The total RNA was treated with DNase I to remove genomic DNA contamination. For reverse transcription, we used an iScript cDNA synthesis kit, which contains random primers, to obtain as representative an RNA mold as possible. The expression of *TcAMT* was quantified by qRT-PCR. PCR was performed using a CFX96 Touch real-time PCR detection system and set up in white opaque polypropylene wells (LightCycler 480 multiwell plate 384) in a final volume of 10 µl per reaction. Briefly, 1 µl of sample DNA (100 ng/µl) was added to 5 µl of a master mix iQ SYBR Green supermix and 4 µl of nuclease-free water with primers 13 and 14, described in Table S1, at a final concentration of 500 nM. Activation of the polymerase was performed at 95°C for 2 min. The PCR program included 39 cycles of denaturation at 95°C for 10 s, annealing, and extension at 59°C for 30 s. SYBR Green fluorescent emission was measured at the end of the elongation step. Subsequently, a melting curve program with a continuous fluorescent measurement starting at 65°C and ending at 95°C (ramping rate of 0.1°C/s) was applied. In order to normalize the expression of *TcAMT*, as housekeeping genes, we used primers for the glyceraldehyde-3-phosphate dehydrogenase (GAPDH) and the ribosomal P0 and L3 genes (primers 15 to 20) (Table S1). The samples were quantified according to the percent threshold cycle (*C*_*T*_) method, and all the assays were performed at least three times.

### Hyposmotic stress.

*TcAMT*-KO and wild-type epimastigotes at log phase of growth were collected by centrifugation at 1,700 × *g* for 10 min, washed twice in PBS, and resuspended at a density of 1 × 10^8^/ml in isosmotic buffer (64 mM NaCl, 4 mM KCl, 1.8 mM CaCl_2_, 0.53 mM MgCl_2_, 5.5 mM glucose, 150 mM d-mannitol, 5 mM HEPES-Na, pH 7.4). The osmolarity of the buffer was adjusted to 300 ± 5 mosM as verified by an Advanced Instruments 3D3 osmometer (Norwood, MA). Hyposmotic stress was induced by a 1:1 dilution of the cell suspension with sterile deionized water, resulting in a final osmolarity of 150 mosM. Cells were centrifuged at 1,700 × *g* for 10 min 30 min after dilution and analyzed by qRT-PCR and Western blotting. To monitor the regulatory volume decrease after hyposmotic stress, the cells were subjected to hyposmotic stress, as described above, and absorbance changes at 550 nm were recorded every 6 s for 10 min using a SpectraMax M2e plate reader (Molecular Devices).

### Starvation assay.

Epimastigotes were washed twice with PBS and collected by centrifugation at 1,700 × *g* for 10 min. The cells were resuspended at a density of 1 × 10^7^/ml in PBS or LIT medium and left for 20 h at 28°C. For Western blot and qRT-PCR assays, aliquots were taken prior to and after incubation for 20 h. Expression of the TcATG8.1 autophagy marker and autophagosome formation in *T. cruzi* epimastigotes incubated in LIT medium or under starvation conditions was estimated by immunofluorescence analysis using anti-TcATG8.1 antibody as described previously (14).

### Preparation and maintenance of oocytes.

*Xenopus laevis* oocytes were purchased from Xenoocyte (Dexter, MI) and used as the standard heterologous expression system for the study of *TcAMT*. Stage IV to V surgically collected oocytes were manually defolliculated and devitellinized with collagenase (1 mg/ml) for 1 h at room temperature and then maintained in filtered Barth’s solution [containing 88 mM NaCl, 1 mM KCl, 0.82 mM MgSO_4_, 0.41 mM CaCl_2_, 2.4 mM NaHCO_3_, 0.33 mM Ca(NO_3_)_2_, and 10 mM HEPES plus 50 µg/ml gentamicin, pH 7.4] at a density of less than 100 per 60-mm plastic petri dish. Barth’s solution was replaced daily.

### cRNA production.

The full-length *TcAMT* open reading frame (ORF) was amplified with Q5 DNA polymerase (Clontech) from *T. cruzi* genomic DNA by PCR using the corresponding gene-specific primers flanking a T_7_ promoter, Kozak consensus sequence, or poly(T)_30_ sequence as indicated in Table S1 (primers 21 and 22). The PCR products were gel purified using a QIAquick gel extraction kit (Qiagen) according to the manufacturer’s instructions, and the double-stranded nucleotide sequences were confirmed by sequencing at GENEWIZ (Research Triangle Park, NC). cRNAs were obtained by *in vitro* transcription using the purified PCR products as the templates with an mMESSAGE mMACHINE kit (Ambion Life Technologies, Thermo Fisher Scientific, Inc., Waltham, MA) according with the manufacturer’s protocol and verified by denaturing agarose gel electrophoresis and ethidium bromide staining.

### cRNA injection.

A horizontal Flaming/Brown micropipette puller (P-97; Sutter Instruments, CA) was used to prepare injection capillaries with a tip resistance of 3 to 4 MΩ. Tips of capillars were then polished with Micro Forge MF-830 (Narishige, Japan) to reach a 10- to 30-μm tip diameter for cRNA injection. Capillars were then backfilled with sterile mineral oil and filled with 3 to 5 μl of cRNA solution. Forty nanograms of cRNA (41.4 to 50.6 nl) per oocyte was injected using the Nanoject II system (Drummond Scientific, CO) and incubated for 72 h at 19°C. Control oocytes were injected with same amount of diethylpyrocarbonate (DEPC) water.

### Electrophysiology.

A standard two-electrode voltage clamp technique was used as previously described (25). Briefly, oocytes were placed in a small-volume (200-µl) perfusion chamber (RC-3Z; Warner Instruments, Hamdem, CT) and superfused with ND96 solution at room temperature. Intracellular recording electrodes made of borosilicate glass capillars with a tip resistance of ~3 MΩ were backfilled with 3 M KCl. For the best temporal resolution and minimization of errors, KCl-agarose bridges connected with Ag-AgCl ground electrodes were used. TcAMT activities were registered with an oocyte clamp (OC-725 amplifier; Warner Instruments, CT). Recordings were filtered at 500 Hz, digitized at a 16-bit 5 kHz using a Digidata 1440 (Axon Instruments, Molecular Devices), and analyzed using pCLAMP 10 software (Axon Instruments). Oocytes were clamped at a −60 mV holding potential. Steady-state currents were recorded in the range from −180 to +180 mV for a 1-s period with 20 mV steps and a 20-s period between steps. Each experiment was done with at least 4 oocytes from 2 different frogs. Oocytes with resting membrane potential (RMP) at the end of experiment that was lower or equal to those at its start were analyzed. All recordings were obtained at room temperature. Oocytes were bathed in ND96 buffer bath solution (containing 96 mM NaCl, 2 mM KCl, 5 mM MgSO_4_, 1 mM CaCl_2_, and 2.5 mM HEPES, pH 7.5) with a continuous perfusion speed of ~2 ml/min. The required pH of ND96 was adjusted with either NaOH or HCl.

### Statistical analysis.

All values are expressed as means ± standard errors (SE). Significant differences between treatments were compared using an unpaired Student *t* test. Differences were considered statistically significant at a *P* of <0.05, and *n* refers to the number of experiments performed. All statistical analyses were conducted using GraphPad Prism 5 (GraphPad Software, Inc., San Diego, CA).
