# Supplementary figures and images for: An Intracellular Ammonium Transporter Is Necessary for Replication, Differentiation, and Resistance to Starvation and Osmotic Stress in Trypanosoma cruzi
Source: mSphere. 2018 Jan 17;3(1):e00377-17. doi: 10.1128/mSphere.00377-17 (PMC5770540; doi:10.1128/mSphere.00377-17)

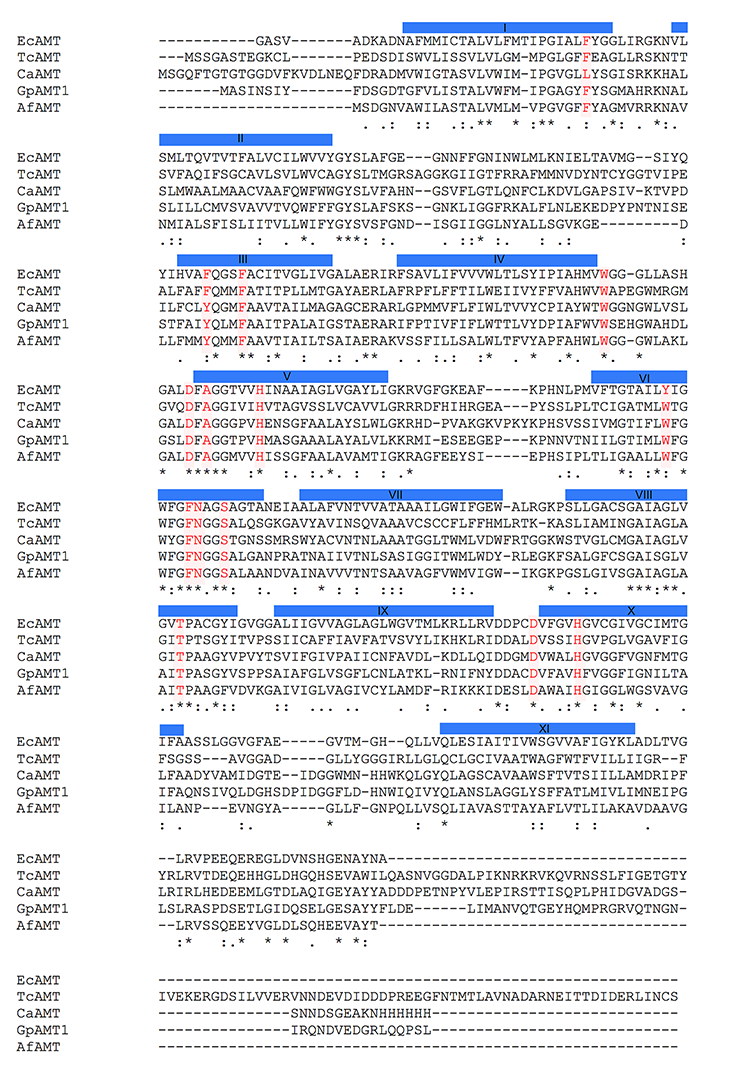

Supplement: FIG S1 [file sph001182453sf1.tif]
