# Supplementary material for: An Intracellular Ammonium Transporter Is Necessary for Replication, Differentiation, and Resistance to Starvation and Osmotic Stress in Trypanosoma cruzi
Source: mSphere. 2018 Jan 17;3(1):e00377-17. doi: 10.1128/mSphere.00377-17 (PMC5770540; doi:10.1128/mSphere.00377-17)
Supplement: TABLE S1 [file sph001182453st1.docx]

|  | Primer | Sequences (5’→ 3’) |  |
| --- | --- | --- | --- |
| 1 | **Fw-OE-AMT** | TTGTCTAGAATGTCTTCCGGCGCCAGTAC |  |
| 2 | **Rv-OE-AMT** | GATAAGCTTTCAAGCGTAATCTGGAACATCGTATGGGTAGCTGCAATTGATTAGCCGTTC |  |
|  |  |  |  |
| 3 | **SgAMT-ET** | GATCGGATCCTTACTACCGATATTGACGAAGTTTTAGAGCTAGAAATAGC |  |
| 4 | **Fw-AMT-Ctag-ultramer** | TGACGAAGTTGACATTGATGACGACCCTCGCGAAGAGGGTTTTAATACAATGACTTTGG  CAGTGAATGCTGATGCAAGGAATGAGATTACTACCGATATT GGTACCGGGCCCCCCCTCGAG |  |
| 5 | **Rv-AMT-Ctag-ultramer** | AAAAGCAAAAGAGTCAAAATAAAAACATTTCGTTTTTGTTTACTTCTCATAAAAATTTACTC  GGCTTGCAGCTGCAATTGATTAGTGGCGGCCGCTCTAGAACTAGTGGAT |  |
| 6 | **Fw-Check-ctag** | AGCAGCCTGTTTATTGGAGAAACTG |  |
| 7 | **Rw-Check-ctag-ko** | AGTGGAATACTAGCACACGCGA |  |
|  |  |  |  |
| 8 | **SgAMT-KO** | GATCGGATCCATTGTGTGGCTTATGTCTTCGTTTTAGAGCTAGAAATAGC |  |
| 9 | **SgRNA-R** | CAGTGGATCCAAAAAAGCACCGACTCGGTG |  |
| 10 | **Fw-AMT-KO-ultramer** | AGCGGCTTCTTTTTATTTTTTATTTTTTCCTTCGGTATCCGGCGTAGGGGAGGGGCGGGGCG  ATAGAAGGGTCGTTTTTGTATTTGCAATTGTGTGGCTTATGGCCAAGCCTTTGTCTCAAGA |  |
| 11 | **Rv-AMT-KO-ultramer** | AAAAGCAAAAGAGTCAAAATAAAAACATTTCGTTTTTGTTTACTTCTCATAAAAATTTACTCG  GCTTTCAGCTGCAATTGATTAGTTAGCCCTCCCACACATAAC |  |
| 12 | **Fw-check-KO** | GCGCTCGAGAGCTTTTTTCTTCTATA |  |
|  |  |  |  |
| 13 | **Fw-AMT-RT** | ATATTCAGTGGATGTGCCGTTCTTTCC |  |
| 14 | **Rv-AMT-RT** | CCGATAATACCTTTACCTCCTGCC |  |
| 15 | **GAPDH-F** | AGCGCGCGTCTAAGACTTACA |  |
| 16 | **GAPDH-F** | TGGAGCTGCGGTTGTCATT |  |
| 17 | **P0-F** | CCTTCTTCCAGGCACTGAAC |  |
| 18 | **P0-R** | AGTTGTCCACACGATCACCA |  |
| 19 | **L3-F** | TGGCTTTGTTGGCTACGGTAC |  |
| 20 | **L3-R** | CCACTGGCTCTTCTCCTTCTTTG |  |
|  |  |  |  |
| 21 | **Fw-AMT-T7** | GAATTGTAATACGACTCACTATAGGGAGACCACCATGTCTTCCGGCGCCAGTAC |  |
| 22 | **Rv-AMT-PolyA** | TTTTTTTTTTTTTTTTTTTTTTTTTTTTTTTCAGCTGCAATTGATTAGCCGTTC |  |
